# The Cytogenetic Profile of Primary and Secondary Plasma Cell Leukemia: Etiopathogenetic Perspectives, Prognostic Impact and Clinical Relevance to Newly Diagnosed Multiple Myeloma with Differential Circulating Clonal Plasma Cells

**DOI:** 10.3390/biomedicines10020209

**Published:** 2022-01-19

**Authors:** Stefanos I. Papadhimitriou, Evangelos Terpos, Konstantinos Liapis, Dimitrios Pavlidis, Theodoros Marinakis, Efstathios Kastritis, Meletios-Athanasios Dimopoulos, Ourania E. Tsitsilonis, Ioannis V. Kostopoulos

**Affiliations:** 1 Department of Laboratory Hematology, Athens Regional General Hospital “Georgios Gennimatas”, 11527 Athens, Greece; sipapadhimitriou@gmail.com (S.I.P.); dimispad@hotmail.com (D.P.); 2 Department of Clinical Therapeutics, School of Medicine, National and Kapodistrian University of Athens, 11528 Athens, Greece; eterpos@med.uoa.gr (E.T.); ekastritis@med.uoa.gr (E.K.); mdimop@med.uoa.gr (M.-A.D.); 3 Department of Haematology, University Hospital of Alexandroupolis, Democritus University of Thrace Medical School, 68100 Alexandroupolis, Greece; kosliapis@hotmail.com; 4 Department of Clinical Hematology, Athens Regional General Hospital “Georgios Gennimatas”, 11527 Athens, Greece; tpmarin1@otenet.gr; 5 Department of Biology, School of Sciences, National and Kapodistrian University of Athens, Panepistimiopolis, Ilissia, 15784 Athens, Greece; rtsitsil@biol.uoa.gr

**Keywords:** primary plasma cell leukemia, secondary plasma cell leukemia, FISH, cytogenetics, clonal evolution, circulating plasma cells, multiple myeloma

## Abstract

Plasma cell leukemia (PCL) is a rare and aggressive plasma cell dyscrasia that may appear as de-novo leukemia (pPCL) or on the basis of a pre-existing multiple myeloma (MM), called secondary plasma cell leukemia (sPCL). In this prospective study, we have applied a broad panel of FISH probes in 965 newly diagnosed MM (NDMM) and 44 PCL cases of both types to reveal the particular cytogenetic differences among the three plasma cell dyscrasias. In order to evaluate the frequency and patterns of clonal evolution, the same FISH panel was applied both at diagnosis and at the time of first relapse for 81 relapsed MM patients and both at MM diagnosis and during sPCL transformation for the 19 sPCL cases described here. pPCL was characterized by frequent *MYC* translocations and t(11;14) with a 11q13 breakpoint centered on the *MYEOV* gene, not commonly seen in MM. sPCL had a higher number of FISH abnormalities and was strongly associated with the presence of del(17p13), either acquired at the initial MM stage or as a newly acquired lesion upon leukemogenesis in the context of the apparent clonal evolution observed in sPCL. In clinical terms, sPCL showed a shorter overall survival than pPCL with either standard or high-risk (t(4;14) and/or t(14;16) and/or del(17p13) and/or ≥3 concomitant aberrations) abnormalities (median 5 months vs. 21 and 11 months respectively, *p* < 0.001), suggesting a prognostic stratification based on cytogenetic background. These observations proved relevant in the NDMM setting, where higher levels of circulating plasma cells (CPCs) were strongly associated with high-risk cytogenetics (median frequency of CPCs: 0.11% of peripheral blood nucleated cells for high-risk vs. 0.007% for standard-risk NDMM, *p* < 0.0001). Most importantly, the combined evaluation of CPCs (higher or lower than a cut-off of 0.03%), together with patients’ cytogenetic status, could be used for an improved prognostic stratification of NDMM patients.

## 1. Introduction

Plasma cell leukemias (PCL) are a rare form of lymphoid malignancies accounting for about 0.3% of leukemias and 0.5–4% of plasma-cell dyscrasias (PCD), with an overall incidence of 0.04 new cases/100.000 individuals per year in Europe [1,2,3]. Traditionally, the diagnosis of PCL has been based on Kyle’s criteria, referring to the presence of >20% plasma cells in the peripheral blood (PB) and/or a circulating plasma cell count of >2 × 10^9^/L [4]. However, there is a debate about these arbitrary thresholds, with several studies suggesting that less strict cut-offs could be used to stratify patients into distinct prognostic subgroups [5,6,7]. Recently, it was proposed that PCL should be defined by the presence of >5% circulating plasma cells based on findings showing the same adverse outcomes as those with the traditional 20% cut-off definition [8]. In most cases (50–70%), PCL appears as a de-novo leukemia, called primary PCL (pPCL), but it can also arise on the basis of a pre-existing, and usually end-stage, multiple myeloma (MM), called secondary PCL (sPCL). To date, the biological mechanisms and the responsible molecular events underlying this leukemic transformation have been inadequately explained.

PCL presents with unique features and a more aggressive clinical behavior when compared to MM. These differences include distinct molecular (both genetic and gene-expression profile), phenotypic and bone marrow (BM) microenvironmental features, a distinct distribution and proportion of cytogenetic abnormalities, higher tumor mass, extramedullary involvement, impaired renal function, increased lactate dehydrogenase (LDH) and β2-microglobulin, and more pronounced anemias and thrombocytopenias [3,5,9,10,11,12]. Moreover, several differences have also been reported between pPCL and sPCL, thus indicating that they should probably be evaluated as distinct clinical entities [13,14,15]. Nevertheless, despite the existence of unifying clinical and molecular features in each PCD, both MM and PCL are characterized by significant heterogeneity, which may confer to a differential prognosis. Current prognostication systems are particularly useful but fail to incorporate the whole variability spectrum, thus necessitating the identification of improved risk-stratification models. Due to its rarity, the genetic background of PCL is not fully elucidated and the limited reported series have mainly included pPCL patients and only a few sporadic sPCL cases. In this study, we performed detailed cytogenetic analyses in a representative series of both pPCL and sPCL, utilizing an extensive panel of probes for all major abnormalities described in MM, and report a different genetic background between the two PCL types. In addition, the prospective evaluation of a large cohort of MM patients with long-term sequential monitoring and follow-up allowed us to monitor the genetic changes occurring gradually during the course of the disease. Our analyses provide new evidence on the cytogenetic background of PCL and highlight cytogenetic clonal evolution to be highly associated with the leukemic transformation of MM to sPCL. Finally, utilizing the highly-sensitive next-generation flow (NGF) approach, we evaluated the presence of clonal circulating plasma cells (CPCs) in a large series of newly-diagnosed MM patients (NDMM) and suggest a model, based on both cytogenetics and CPCs, capable of stratifying patients into distinct prognostic groups. 

## 2. Patients and Methods

### 2.1. Patients

The study included 965 NDMM and 44 PCL patients who were diagnosed and treated between January 2005 and May 2020. PCL was considered when both of Kyle’s criteria were met, namely when a patient was found with both >20% clonal plasma cells in PB and a circulating plasma cell count higher than 2 × 10^9^/L. Among PCL patients, 25 patients were diagnosed with de-novo leukemia (pPCL) and 19 patients developed leukemia during MM progression (sPCL) with a median time interval for the leukemic transformation of 15 months (range: 6–56 months). The clinical characteristics of all patients are presented in Table 1. 

Patients were tested at diagnosis (of MM or pPCL) for the presence of major cytogenetic aberrations described in MM (described in detail below in Section 2.3). The same analysis was also performed in 81 relapsed MM patients including 19 patients with sPCL transformation, in order to highlight the genetic changes occurring during disease progression (see Section 2.3 for details). 

PCL patients were treated with various regimens. NDMM patients, evaluated for the presence of CPCs (n = 161, see below for details), were treated with either bortezomib, lenalidomide, dexamethasone (VRD) or bortezomib, cyclophosphamide, or dexamethasone (VCD) as induction regimens followed by high-dose melphalan (HDM) with autologous stem cell transplantation (ASCT), according to the protocol described in detail elsewhere [16]. The number of CPCs and the proportion of patients bearing high-risk aberrations were equally distributed between the two treatment groups (data not shown). The study was approved by the local ethics committee and patients signed informed consent according to the Declaration of Helsinki. 

### 2.2. Purification of Plasma Cells from BM and PB Samples

Plasma cell enrichment was performed in each BM and PB sample prior to FISH analysis (described in next paragraph). In brief, 3–5 mL of BM and/or PB were used for the osmotic lysis of erythrocytes using NH4Cl-lysis buffer and the acquired cells were washed with phosphate-buffered saline (PBS) containing 0.5% fetal bovine serum. The isolation of plasma cells was subsequently performed with the positive immunomagnetic selection of CD138^+^ cells using human CD138 magnetic microbeads (MACS, Miltenyi Biotech, Bergish Gladbach, Germany) following the manufacturer’s instructions. The density and viability of isolated cells were evaluated in a hemocytometer with Trypan Blue 0.4% and the purity of plasma cells was tested by flow cytometry after staining for CD38-FITC and CD138-PerCP. When CD38^+^CD138^+^ cells constituted less than 90% of the isolated material, the immunomagnetic separation procedure was repeated. Purified plasma cells were then laid on polylysine-coated slides after cytocentrifugation and fixed as previously described [17]. A minimum number of 250 cells were available for FISH evaluation in all cases.

### 2.3. Interphase Fluorescent In-Situ Hybridization (i-FISH)

Purified plasma cells of each patient were evaluated by i-FISH for the presence of major cytogenetic abnormalities described in MM. In particular, commercially available probes (mostly Abbott Molecular, Des Plaines, IL, USA) were used for the detection of del(13q), t(14q32), t(11;14), t(4;14), t(14;16), −17/del(17p13), del(1p32), +1q21, t(8q24), and hyperdiploidy (HD). HD was considered when a minimum of three chromosomes were overrepresented without any evidence of monosomy. For this, a probe set targeting chromosomes 5, 9, and 15—the most commonly overrepresented chromosomes in HD [18,19]—was applied in each patient, and, when needed, additional centromeric probes were also used to verify numerical aberrations indirectly indicated by the application of all other probes. High-risk cytogenetics was considered the presence of t(4;14) and/or t(14;16) and/or del(17p13) and/or t(8q24) and/or the presence of at least three concomitant abnormalities (other than trisomies) of all aberrations tested. In a different case, a patient was considered as having a standard-risk status.

For t(11;14) evaluation, two independent dual fusion probes were applied, both containing the same *IGH* probe but different *CCND1* probes targeting sequences on the 11q13.3 location. Multiple 13q probes were also used for a more detailed characterization of the 13q deleted region of chromosome 13, as described in detail in the next paragraph. The cut-off levels for each probe were set as the mean +3x standard deviation of positive values reached upon analysis of CD138^+^ isolated cells from healthy donors. A summary of all probes used with the specific cytogenetic location targeted and the estimated cut-off levels are presented in Supplementary Table S1.

As mentioned above, the FISH examination was performed in all patients at the time of MM and pPCL diagnosis and at the time of first relapse (based on criteria set by Rajkumar et al. [20]) in 62 MM patients and sPCL transformation for the 19 sPCL cases included in the study. This cytogenetic re-evaluation allowed for the detection of clonal evolution as a consequence of disease progression. Therefore, clonal evolution was considered as the presence of a new, acquired abnormality (in relapsed and sPCL patients) that was absent or below the cut-off levels at the initial diagnostic MM evaluation. The median time interval between the initial FISH examination (on MM diagnosis) and the sequential re-evaluation in the relapsed MM cases were 25 months (range: 6–69 months) and 15 months (range: 6–56 months) for those patients showing a leukemic transformation into sPCL. 

### 2.4. 13q Deletions

The presence of 13q deletions was investigated with the use of probes targeting the 13q14 locus (D13S319, D13S272, D13S25 and RB1), together with a probe for 13q34 and 13q terminal region (13qter) to distinguish between interstitial and terminal deletions. (Supplementary Figure S1). A terminal deletion was considered when a single fluorescent signal per cell was detected for each of these six applied probes. This mapping process was performed in all PCL cases and in 280 randomly selected MM cases at diagnosis for comparative reasons. 

### 2.5. t(11;14)

In all PCL cases and 275 NDMM patients, t(11;14)(q13;q32) was investigated with the application of two similar but distinct probe sets. Both contained a green probe targeting the *IGH* gene on the 14q32 region. The first one used a 378 kb probe spanning the entire length of *CCND1* gene on 11q13 and extending in both the centromeric and telomeric directions. The second probe set (XT) used a much larger red probe (850 kb) centered on the *MYEOV* gene. Hence, a breakpoint close to *CCND1* would produce two fusion signals with both sets, while a breakpoint close to *MYEOV* would produce two fusion signals with the application of the XT probe set (signal pattern:2F1G1R and a single fusion with the application of the first probe set (signal pattern 1F2G1R).

### 2.6. Detection of Circulating Plasma Cells (CPCs) with Next-Generation Flow Cytometry (NGF) 

The presence of clonal CPCs was evaluated in the PB samples of 161 NDMM with the NGF protocol for the detection of minimal residual disease (MRD) according to the EuroFlow guidelines [21,22,23]. Cells were lysed with the bulk-lysis protocol and the acquired cells were stained with the proposed eight-color panels for the detection and efficient discrimination of clonal plasma cells of any phenotype from the remaining PB nucleated cells. A minimum number of six million events were acquired per sample, for a median limit of detection (LOD) reached of 3.5 × 10^−6^ and a median limit of quantification (LOQ) reached of 8.8 × 10^−6^, setting 20 and 50 cells, respectively, as a prerequisite cut-off point for the relevant measures. Samples were acquired in a FACSCantoII cytometer (BD Bioscience, San Jose, CA, USA) operating with the optimal photomultiplier (PMT) voltages and settings based on the EuroFlow standard operating procedure (SOP) for instrument set-up. Data analysis and the evaluation of CPC detection was performed with the Infinicyt software (version 2.0, Cytognos, S.L., Salamanca, Spain).

### 2.7. Statistical Analysis

All statistical analyses were performed with the Statistical Package for the Social Sciences software v.20 (IBM SPSS Statistics, Inc., Chicago, IL, USA). Differences in binomial variables among groups were evaluated with the chi-square test, while one-way ANOVA or the non-parametric Kruskal–Wallis approach were selected for assessing differences in continuous variables depending on the distribution of their values. Chi-square contingency table analyses were also assessed to examine for possible intercorrelations between the cytogenetic abnormalities, and logistic regression analysis was performed to predict the risk of developing pPCL and/or sPCL based on the available baseline characteristics of all NDMM and PCL patients described here. The optimal cut-off point of CPCs for discriminating NDMM patients with either high or standard-risk cytogenetics was selected on the basis of maximum specificity and sensitivity values obtained by the relative receiver operating characteristic (ROC) curve. The survival analyses were assessed using standard Kaplan–Meier curves and log-rank statistics. All analyses were two-sided and statistical significance was assumed at *p* < 0.05.

## 3. Results

### 3.1. Clinical Characteristics of PCL and NDMM Patients

Compared to NDMM, PCL patients of both types had a younger age of disease onset (median: 60 years old for pPCL vs. 65 for sPCL vs. 68 for NDMM), a higher BM plasma cell infiltration rate (median: 70% for pPCL and 80% for sPCL vs. 55% for NDMM, *p* < 0.001 for NDMM vs. both PCL types) and a higher incidence of extramedullary involvement (median: 20% for pPCL and 27% for sPCL vs. 8% for NDMM, *p* < 0.5 for NDMM vs. both PCL types) (Table 1). Moreover, PCL patients suffered more frequently from anemia and/or thrombocytopenia and had elevated LDH levels (median: 330 U/L for pPCL and 232 for sPCL vs. 175 for NDMM, *p* < 0.001 for NDMM vs. both PCL types). No differences were observed between the three PCDs in serum albumin or calcium levels and the immunoglobulin heavy or light chain isotype.

Particular differences were also observed between the two PCL types. The presence of bone lytic lesions was more apparent in sPCL than in pPCL (68.8% in sPCL vs. 45.4% in pPCL; *p* = 0.19), which is consistent with sPCL originating from NDMM, in which lytic lesions occur in approximately 2/3 of patients. The median concentration of β2-microglobulin was significantly different between the three groups of patients, with the higher values observed in pPCL (7.3 mg/L in pPCL vs. 3.8 in sPCL and 3.3 in NDMM; *p* < 0.0001). Similarly, serum creatinine levels were increased (>2.0 mg/dL) in 36.0%, 21.1% and 14.3% of pPCL, sPCL and NDMM patients, respectively, thus indicating different levels of renal dysfunction between in the three disorders (Table 1).

### 3.2. pPCL and sPCL Patients Have Distinct Cytogenetic Profiles

The detailed cytogenetic pattern of PCL patients evaluated by i-FISH is shown in Table 2. Compatible with a more aggressive clinical course, PCL was presented with a significantly higher frequency of aberrations when compared to NDMM. In particular, the mean number of cytogenetic findings per sample was found to be 1.4, 2.9 and 3.9 for NDMM, pPCL and sPCL, respectively. pPCL patients had at least one aberration and all sPCL cases were found with a minimum of two concomitant aberrations; contrarily, 15.1% of NDMM patients had no detectable abnormalities. Hyperdiploidy was the only aberration found more frequently in NDMM than in either pPCL or sPCL (52.1% in NDMM vs. 20.0% in pPCL vs. 31.6% in sPCL; *p* < 0.01 for comparing NDMM vs. pPCL).

Beyond the apparent differences with NDMM, our analysis revealed a clearly distinct cytogenetic profile between the two leukemia types. Del(13q) was the most frequent finding for both leukemias, though its relative prevalence was significantly different (15/25 (59.1%) in pPCL vs. 18/19 (94.7%) in sPCL; *p* = 0.013). −17/del(17p13) was mainly found in sPCL (4/25 (16%) in pPCL vs. 13/19 (68.4%) in sPCL; *p* < 0.001), whereas t(11;14) was detectable only in pPCL, and notably, in the majority of pPCL patients (13/25 (52%) in pPCL vs. 0/16 (0%) in sPCL; *p* < 0.001). t(4;14) and chromosome 1 aberrations were more prevalent in sPCL, although the differences from pPCL were not statistically significant, probably due to the relatively limited number of cases in each group. On the other hand, the prevalence of *MYC* translocations was higher in pPCL, 1.5 times more frequent than in sPCL [10/25 (40%) in pPCL vs. 5/19 (26.3%) in sPCL; *p* = 0.21] and four times more frequent than in NDMM [24/265 (9.1%); *p* < 0.0001]. 

The contingency analysis showed a strong positive correlation between the presence of −17/del(17p13), t(4;14) and 13q deletions (in all binary combinations) in both NDMM and pPCL; all four pPCL cases positive for del(17p13), and respectively, all four pPCL cases positive for t(4;14)—two patients had concomitant 17p—and t(4;14)- were also positive for extended 13q deletions. On the other hand, the presence of t(11;14) showed a negative association with 13q- in NDMM, as only 16/76 (21.1%) patients with t(11;14) were also positive for 13q-; of note, this correlation was completely reversed in pPCL, where 9/13 (69%) patients with detectable t(11;14) showed also a concomitant 13q deletion. 

### 3.3. 13q Deletions in NDMM and PCL Patients

The length of 13q deletions was evaluated with the employment of six FISH probes targeting different chromosomal 13q loci. Our results showed an extended 13q deletion in all 33 PCL cases with detectable del(13q) (the application of each probe returned a single fluorescent signal). Following the same process for NDMM patients, we found that among 115 cases with del(13q) (and tested with all different 13q probes), only 94 (81.7%) showed evidence of an extended 13q-. The remaining 21 cases had interstitial deletions, as depicted in Supplementary Figure S1. In particular, 18 patients had a deletion restricted only in the 13q14 locus (two of them only in the 13q14.3 region), and two patients showed a broad deletion of the long arm, yet not terminal, since the 13qter region was found intact. Finally, in one NDMM patient, the deleted area was restricted only in the peripheral part of the 13q arm, as indicated by the absence only of 13q34 and the 13q telomeric region.

### 3.4. Unique Cytogenetic Pattern of t(11;14)(q13;q32) in pPCL

t(11;14)(q13;q32) is the most common translocation in MM and is considered as a standard-risk cytogenetic marker with a more favorable outcome when compared with other *IGH* translocations and/or del(17p13). However, t(11;14) is the most common genetic lesion in the aggressive pPCL, which may probably indicate a different molecular background. In this context, we concurrently utilized two different dual-fusion probes targeting t(11;14), in an effort to gain a better insight of the cytogenetic pattern of this rearrangement in the two dyscrasias. 

In both NDMM and pPCL, the application of the two independent probes resulted at the same FISH result for each patient (either positive or negative). The majority of NDMM patients who were found positive for t(11;14) (35/40 tested with both probes, 87.5%) showed the same signal pattern on the two different probes, even in those cases with an “atypical” signal formation type (Supplementary Table S2). On the contrary, half of t(11;4) positive pPCL patients (6/13, 46.2%) showed dissimilar signal patterns in the two probes; the application of the smaller probe resulted in the detection of only one fusion signal (1F2G1R), while the larger XT probe showed the typical formation of two fusion signals (2F1G1R), thus implying a non-typical 11q13.3 breakpoint close to the *MYEOV* gene within a region delimited by a 30 kb point upstream the *MYEOV* extending 425 kb towards the centromere based on the probe map. These sequences in the 11q arm were detectable only with the larger FISH probe and host various non-coding RNA genes and five protein-coding genes with multiple structural or regulatory functions (Supplementary Table S3).

### 3.5. Clonal Evolution by Sequential Cytogenetic Analysis during Disease Progression

**Figure 1:**
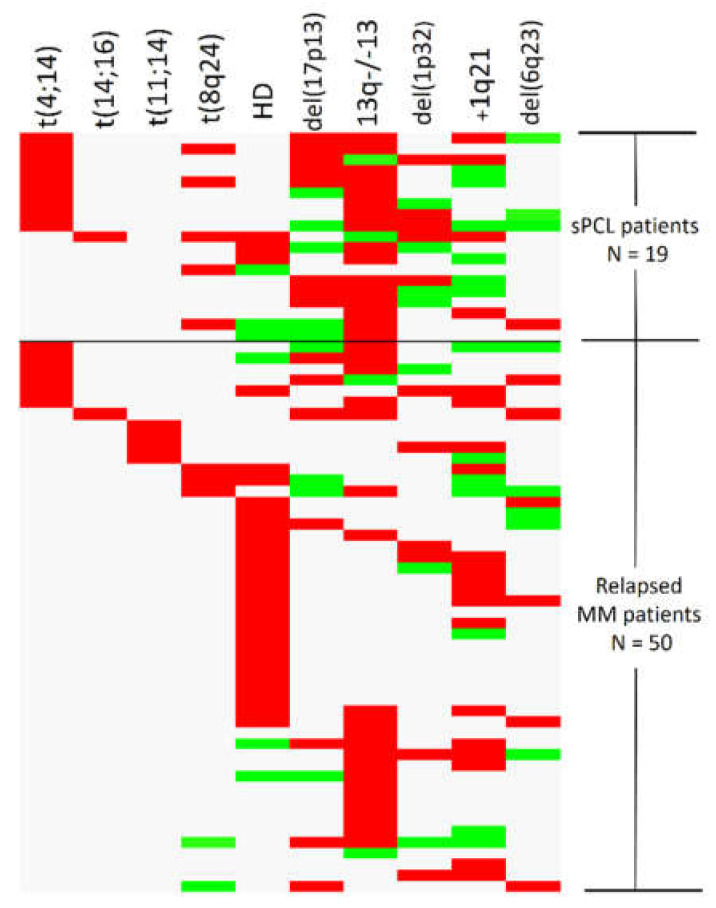
Clonal evolution as detected by the acquisition of novel abnormalities during multiple myeloma (MM) progression. Each row represents the cytogenetic pattern of a single patient. The baseline aberrations detected at initial FISH examination during MM diagnosis are shown in red, whereas the new acquired aberrations during plasma cell leukemia transformation (sPCL, upper part) or at relapse (bottom part) are shown in green.

The cytogenetic re-evaluation of NDMM transforming into sPCL revealed novel acquired aberrations for the majority of patients. The baseline abnormalities were present in all patients during FISH re-evaluation, with 17/19 (89.4%) sPCL cases showing clear evidence of clonal evolution via a median of 1.16 newly acquired abnormalities per case. On the contrary, clonal evolution was evident in only 18/62 (29%) relapsed MM patients with a ratio of 0.42 new acquiring abnormalities per case (Figure 1).

Overall, clonal evolution was more prevalent in patients with the most complex baseline cytogenetics (i.e., ≥2 concurrent aberrations); indeed, only 2/17 (11.8%) sPCL and 5/18 (27.8%) relapsed MM patients with apparent clonal evolution had a single chromosomal abnormality at diagnosis. Moreover, there was only 1/13 (7.7%) NDMM patients with no baseline aberrations (or aberrations at undetectable levels below their relevant cut-off, referred to in Supplementary Table S1), who showed evidence of clonal evolution on relapse. Del(17p13) was the most frequent abnormality implicated with detectable clonal evolution in both sPCL and relapsed MM, either when present as a baseline abnormality or as a newly acquired one upon disease progression. Similarly, *MYC* translocation was commonly involved in clonal evolution process, always as a baseline feature in sPCL, but also as a secondary lesion in relapsed MM. Deletions of 13q were already present at the early myeloma stages, in contrast with chromosome 1 aberrations (mostly 1q amplification) and del(16q23), which were the most frequent novel lesions detected in advanced disease stages. 

### 3.6. Differential Clinical Outcomes among PCL Patients and a Stratification Model for NDMM

To date all but three PCL patients have died, showing a significantly shorter overall survival (OS) than in NDMM. Two pPCL patients are alive after 36 and 42 months, having received VRD and bortezomib, thalidomide, and dexamethasone (VTD) respectively, followed by allogeneic stem cell transplantation in both cases. One sPCL patient is also alive 14 months after leukemia diagnosis, having received bortezomib-based treatment. 

**Figure 2:**
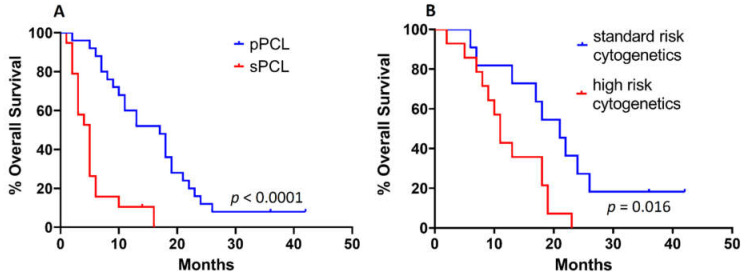
(**A**): Overall survival of patients since the time of their diagnosis with primary and secondary plasma cell leukemia (pPCL and sPCL, respectively). (**B**): Overall survival of pPCL patients according to their cytogenetic status.

The median OS of pPCL was poor (17 months), however significantly better than that of sPCL, reaching a median of only 5 months (*p* < 0.0001) (Figure 2A). Interestingly, at a subgroup level, pPCL patients with standard-risk cytogenetics demonstrated an improved OS with a median of 21 months when compared with their high-risk pPCL counterpart or pPCL patients with a complex karyotype (i.e., three or more aberrations) showing a median OS of 11 months (*p* = 0.016, HR: 2.49, 95% CI: 1.07–5.77, Figure 2B).

Altogether, our observations display the apparent association of PCL with a higher cytogenetic complexity and highlight a clinical stratification of pPCL patients according to their cytogenetic profile. 

**Figure 3:**
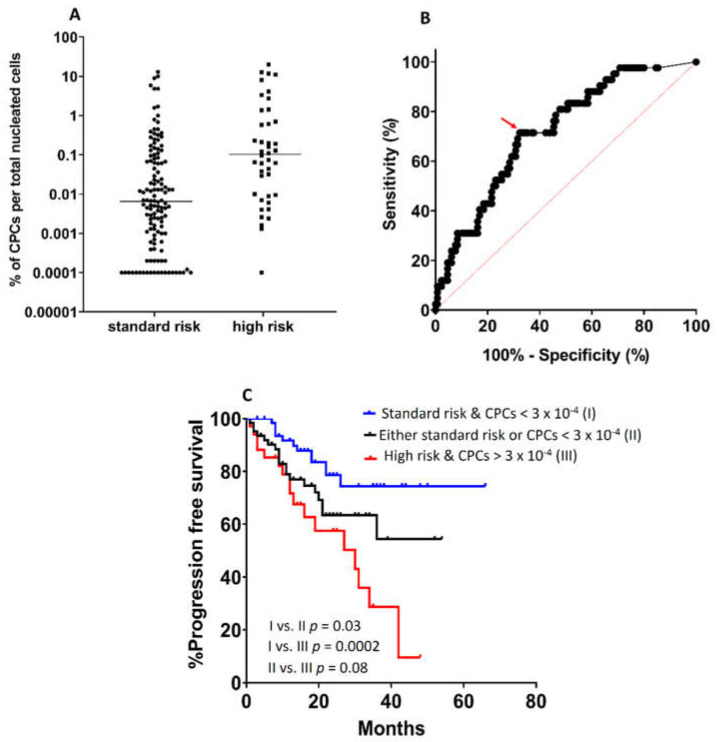
(**A**): Association of cytogenetic status with the presence of circulating plasma cells (CPCs) in newly diagnosed Multiple Myeloma (NDMM) patients. High-risk patients [i.e., with t(4;14) and/or t(14;16) and/or del(17p13) and/or t(8q24) and/or ≥3 concomitant aberrations] have higher numbers of CPCs. (**B**): Receiver operating characteristic (ROC) curve for the estimation of the optimal cut-off value of CPCs (red arrow) discriminating among high-risk and low-risk patients. (**C**): Progression-free survival (PFS) of patients according to their cytogenetic status and the number of CPCs below or higher than 3 × 10^−4^ (% of nucleated cells).

Following the same approach on the NDMM setting, we observed a clear correlation between patients’ CPC levels and their cytogenetic status; patients with high-risk cytogenetics had a median value of 1.1 × 10^−3^ CPCs (% of total nucleated cells) vs. 7 × 10^−5^ for patients with standard-risk abnormalities (*p* < 0.0001, Figure 3A). Using the ROC curves, the optimal cut-off point of CPCs for the cytogenetic discrimination of NDMM patients with >70% sensitivity and specificity was defined as 3 × 10^−4^ (Figure 3B). Importantly, when combined together, the cytogenetic profile and the number of CPCs could stratify patients into three distinct prognostic groups. In particular, the 3-year progression-free survival (PFS) for standard-risk NDMM patients with CPCs below the cut-off was 71% (median PFS, not reached, NR) vs. 55% for patients with either high-risk cytogenetics or high CPCs (median PFS, NR), vs. only 28% for patients with both high-risk aberrations and CPCs > 3 × 10^−4^ (median PFS, 30 months, *p* = 0.03, Figure 3C).

## 4. Discussion

Due to its rarity, a limited series of PCL have been published to date, most of which including relatively small numbers of patients [3,24]. As a consequence, our understanding in the oncogenic mechanisms and the biology leading to the aggressive clinical course of PCL remain elusive. Similarly, the molecular defects of PCL—especially those of the sPCL type- remain inadequately explored, with most of the reported series referring to retrospective studies or case reports, many of which apply conventional cytogenetics that may underestimate some of the major chromosomal aberrations detected by more sensitive approaches, such as 17p deletions and/or some of the 14q32 rearrangements [15,25,26,27,28,29]. In the present study, we have prospectively evaluated a broad panel of abnormalities highlighting particular dissimilarities between NDMM, pPCL and sPCL and further displayed clonal evolutionary patterns associated with the leukemic transformation of primary MM to sPCL. Moreover, our data support the clinical relevance of a differential cytogenetic background in PCL and provide a simple algorithm based on both cytogenetics and CPCs levels for a better patient stratification in the NDMM setting.

As expected, the presentation of PCL showed a more aggressive clinical behavior when compared with NDMM, evidenced by a higher frequency of anemia and thrombocytopenia, higher BM infiltration levels, complex karyotypes and often extramedullary plasmacytomas. Nevertheless, differences were also observed between the two leukemia types, with creatinine and β2-microglobulin levels being significantly higher in pPCL, in agreement with some previous reports [13,26]. Most importantly, cytogenetic analyses revealed a quite dissimilar genetic background in the two PCLs, further supporting the notion that pPCL and sPCL represent distinct biological entities [3,5,30]. 

The frequency of *IgH* translocations was similar in both PCL types and higher than in NDMM; however, the spectrum of the rearranged loci was completely different; t(14;16) was relatively rare in both leukemias, t(4;14) clearly predominated in sPCL, whereas t(11;14) was exclusively present in pPCL, verifying previous studies reporting its higher frequency in pPCL and hence reinforcing the view of a possible etiologic role of this aberration in the pathogenesis of pPCL [4,9,13,31,32,33,34]. The presence of t(11;14) is traditionally regarded as a standard risk marker in MM correlating with a better outcome, though there is new evidence that may refute these stratification models [35]. It is becoming clear that t(11;14) MM is a heterogeneous disease [36] and novel findings show that t(11;14)-positive pPCL and MM have a different genetic and transcriptional background [37,38]. The application of two different t(11;14) probes in our series has revealed an atypical breakpoint in the 11q13 region in almost half of pPCL patients (contrarily to 13% in NDMM), providing evidence for different genetic loci and novel candidate genes that may be implicated in the appearance of the leukemic phenotype (Supplementary Table S3). These data further support the idea of separate t(11;14) molecular defects between MM and pPCL and echo the need for future studies to evaluate the role of these emerging genes in the pathogenesis of pPCL. 

Accordingly, we found a significantly higher predominance of 8q24 rearrangements in pPCL, thus imputing *MYC* deregulation as another possible cause for pPCL ontogeny. The percentage found in our study is higher than that reported by Avet-Loiseau et al. [31] (~10% of pPCL cases when evaluated on metaphases) and Tiedemann et al. [13] (8% or 33% of cases by utilizing different probes) but harmonizes with the results of Chiecchio et al. [39], reporting *MYC* abnormalities in 7/10 pPCL patients when using the 8q24 break-apart probe, which we also applied (Supplementary Table S1). Interestingly, only a small counterpart of these cases were found positive for t(8;14) (3/10 in our study and 1/7 in the British study [39]) implying that *MYC* dysfunction has a pivotal role in the disease phenotype, regardless of the accompanying rearranged locus.

Del(1p32) and 1q amplifications, which are frequent in MM were also found in our PCL cohort with increased rates in the sPCL type. Similarly, del(17p13) and 13q-, which were both more frequent in PCL than MM, had a much higher predominance in the sPCL form, reaching a frequency of 68% and 95%, respectively. Of note, our analysis with various 13q probes revealed an extensive deletion of the 13q arm for PCL patients (of both types) bearing 13q defects (thus showing indirect evidence for monosomy 13),unlike MM, in which about 20% of patients with 13q abnormalities may show interstitial deletions, mainly restricted in the 13q14 region [40,41]. Hyperdiploidy was the only abnormality found more commonly in NDMM than PCL (53% in NDMM vs. 25% in PCL as a whole), consistent with previous reports [13,31,39,42,43], probably reflecting the more favorable outcome commonly seen in HD cases.

The sequential FISH analysis allowed for the identification of novel acquired aberrations during disease progression and thus, tracking clonal evolutionary patterns in both relapsed MM and sPCL. Clonal evolution is a common phenomenon in all malignancies, including MM, which may be manifested either via linearly-related subclones with a homogenous mutational background that accumulate novel genetic lesions during the course of the disease, or, most commonly, via a Darwinian-like branching model, where distinct branches may acquire different and irrelevant genetic abnormalities, leading to a substantial genetic diversity of coexisting dominant and minor subclones with heterogeneous mutational profiles [44,45,46]. The vast majority of patients in our series (89%) showed novel acquired abnormalities at the sPCL stage. This frequency was three-times higher than the relevant incidence of newly detected lesions in the relapsed setting, thus providing strong evidence that clonal evolution accompanies the leukemic transformation of MM, either as a result of new acquired mutations or due to the emergence of “indistinguishable” chemoresistant clones that were present at onset but as minor subclones. The occurrence of clonal evolution was favored by a disadvantageous genetic background (high-risk and/or complex karyotype) at initial diagnosis, but was not limited thereby, since there were also cases where clonal evolution occurred in a pre-existing favorable hyperdiploidic profile. Del(13q) was mostly seen at diagnosis, del(16q) was the most common secondary event, whereas chromosome 1 abnormalities and del(17p) could be detected either at diagnosis or at advanced stages, similarly with previous findings describing the chronological genetic landscape of MM [19,47,48]. Most importantly, though, our findings clearly underscore the association of del(17p13) with secondary plasma cell escape and support the notion that TP53 deregulation could sufficiently explain the aggressiveness and the apparent genomic instability observed in sPCL [49,50].

The distinct cytogenetic profile in the two PCLs, with the apparently higher accumulation of genetic defects in sPCL—especially those with an adverse prognostic impact—could explain the significantly worse clinical course of sPCL. Besides, the presence of cytogenetic clonal evolution, indicative of expanded genomic instability, is known to be associated with inferior outcomes, irrespective of whether the newly acquired lesion is of high risk [48,51,52]. However, the prognostic impact of cytogenetic lesions in pPCL remains a matter of debate. Several studies have reported lack of significant association between cytogenetics and PFS and/or OS [9,34,53,54], whereas Avet-Loiseau et al. [31] and Chang et al. [25] showed that t(11;14) is associated with prolonged OS, and, in another study, t(4;14) was the only aberration with an independent negative prognostic impact on pPCL survival [25]. Moreover, in a large series studied by Pagano et al. [42] the good prognostic karyotype (t(11;14), hyperdiploidy) induced a 37.6-times lower death-risk than pPCL with high-risk cytogenetics, and similarly, Jung et al. reported that del(17p) and a complex karyotype conferred reduced OS in a subset of pPCL patients who had not received conventional chemotherapy [55]. Our data support the survival benefit for pPCL patients with no complex karyotype or high-risk aberrations, showing a reduced death-risk by 2.5 times. However, a profound limitation of our study was the heterogeneous treatment modalities applied in our PCL cohort, which may question the exact clinical impact of these observations. Prospective clinical trials on PCL cohorts (either defined with traditional or the new proposed 5% criteria) are definitely warranted to establish the exact impact of cytogenetics on a common therapeutic background.

The profound association of PCL with a burdened cytogenetic profile and its further distinct background between pPCL and sPCL may firmly explain the differential outcomes of the three PCDs. In this context—and taking into account the heterogeneous outcomes seen for NDMM patients—we evaluated the association of cytogenetics with the differential presence of CPCs in NDMM, whose high number is regarded as a negative prognostic factor with an independent value. Using the sensitive NGF approach for CPC detection, we found a critical cut-off point differentiating patients based on their cytogenetic-risk status. Most importantly, our data highlight a solid model of combining FISH results with CPC numbers for the efficient stratification of ASCT-eligible NDMM patients into three subsets, with a distinct clinical outcome. These approaches could prove particular useful for the early discrimination of ultra-high risk NDMM patients, who could benefit from the administration of a more intensive therapeutic approach. 

Overall, our observations support a distinct genetic background between the two PCL types which may confer to their different clinical course. *MYC* alterations and t(11;14) seem strongly associated with pPCL pathogenesis with a non-classical t(11;14) genetic fingerprint possibly explaining the different molecular events leading to pPCL onset and not to MM, for cases bearing this rearrangement. On the other hand, P53 deregulation, along with its consequent genetic instability, leading to apparent clonal evolution, seems strongly correlated with the leukemic transformation of primary MM to sPCL. On clinical grounds, our findings support a cytogenetic-based stratification of PCL patients, which lead to the evaluation of a simple but efficient prognostic model for NDMM, discriminating patients according to their CPC number and cytogenetic status. Further prospective studies in larger patient cohorts are warranted to reveal the exact molecular mechanisms leading to leukemogenesis and validate the clinical impact of these findings in the NDMM setting.

## Figures and Tables

**Table 1:** Clinical presentation features of PCL and NDMM patients enrolled in the study.

	pPCL n = 25	sPCL n = 19	NDMM n = 965	*p* Value NDMM vs. pPCL	*p* Value NDMM vs. sPCL	*p* Value pPCL vs. sPCL
age (years)	60 (45–81)	65 (42–80)	68 (29–92)	<0.01	n.s.	n.s.
male sex (%)	11/25 (44%)	10/19 (52.6%)	523/965 (54.2%)	n.s.	n.s.	n.s.
lytic lesions	10/22 (45.4%)	11/16 (68.8%)	276/410 (67.3%)	0.04	n.s.	n.s.
extramedulary involvement	4/20 (20%)	4/15 (26.7%)	23/301 (7.6%)	0.07	0.03	n.s.
hemoglobin (g/dL)	8.5 (5.8–13.6)	9.1 (6.8–11.2)	10.4 (4–18)	<0.001	0.002	n.s.
platelets (×10^9^/L)	108 (10–250)	100 (30–300)	244 (22–585)	<0.0001	<0.0001	n.s.
WBC (×10^9^/L)	15 (3.5–40)	15 (4.7–34.5)	6.1 (2.2–70)	<0.0001	<0.0001	n.s.
BM infiltration (%)	70 (30–100)	80 (35–100)	55 (5–100)	<0.001	<0.0001	n.s.
PB plasmacytosis (×10^9^/L)	5.4 (0.9–72)	6.1 (1.2–65)	-	-	-	n.s.
calcium (mg/dL)	9.4 (8.3–14.4)	9.6 (6.5–12.5)	9.5 (6.3–15.5)	n.s.	n.s.	n.s.
LDH (U/L)	330 (100–690)	232 (120–550)	175 (68–860)	<0.0001	<0.001	0.01
serum albumin (g/dL)	3.7 (2.7–4.4)	3.6 (2.5–4.9)	3.9 (1.8–5.1)	n.s.	n.s.	n.s.
creatinine >2 mg/dL	9/25 (36%)	4/19 (21.1%)	101/708 (14.3%)	<0.007	n.s.	n.s.
b-2 microglobulin (mg/L)	7.3 (1.4–11.2)	3.8 (1.7–7.8)	3.3 (0.38–70)	<0.0001	n.s.	<0.0001
M-protein IgG IgA IgD light chain only non-secretory	13/25 (52%) 3/25 (12%) 1/25 (4%) 5/25 (20%) 3/25 (12%)	10/19 (52.6%) 4/19 (21.1%) 1/19 (5.3%) 4/19 (21.1%) 0/16	482/965 (49.9%) 221/965 (22.9%) 33/965 (3.4%) 150/965 (15.5%) 79/965 (8.2%)	n.s.	n.s.	n.s.
kappa light chain	12/20 (60%)	7/15 (46.7%)	569/965 (58.9%)	n.s.	n.s.	n.s.
Phenotype 19+ 45+ 56+ 117+	0/11 2/11 (18.2%) 5/11 (45.4%) 1/11 (9.1%)	N/A N/A N/A N/A	12/445 (2.7%) 80/445 (18%) 281/445 (63.1%) 181/445 (40.7%)	n.s. n.s. n.s. 0.03	N/A	N/A

All values shown for continuous variables are median with ranges in parentheses. pPCL, primary plasma cell leukemia; sPCL, secondary plasma cell leukemia; NDMM, newly-diagnosed Multiple Myeloma; n.s., non-significant; N/A, not applicable; WBC, white blood cells; BM, bone marrow; PB, peripheral blood, LDH, lactate dehydrogenase.

**Table 2:** Frequency of cytogenetic aberrations in the three plasma cell dyscrasias.

Cytogenetic Abnormality	pPCL	sPCL	NDMM	*p* Value
del(13q)	15/25 (59.1%)	18/19 (94.7%)	334/846 (39.5%)	<0.0001 ^a^
t(4;14)	4/25 (16%)	9/19 (47.4%)	92/927 (9.9%)	0.0006 ^a^
t(11;14)	13/25 (52%)	0/19 (0%)	76/542 (14%)	<0.001 ^b^
t(14;16)	2/25 (8%)	1/19 (5.3%)	21/862 (2.4%)	n.s
−17/del(17p13)	4/25 (16%)	13/19 (68.4%)	77/899 (8.6%)	<0.001 ^c^
t(8q24)	10/25 (40%)	5/19 (26.3%)	24/265 (9.1%)	<0.0001 ^d^
del(1p32)	7/25 (28%)	9/19 (47.4%)	42/289 (14.5%)	0.003 ^a^
+1q21	8/25 (32%)	10/19 (52.6%)	191/605 (31.6%)	0.038 ^a^
del(16q23)	4/25 (16%)	4/19 (21.1%)	39/279 (14%)	n.s
Hyperdiploidy	5/25 (20%)	6/19 (31.6%)	151/290 (52.1%)	0.0032 ^d^
Normal (no aberrations)	0/25	0/19	38/252 ^e^ (15.1%)	0.15
Only one aberration	6/25 (24%)	0/19	145/252 ^e^ (57.5%)	<0.001
Average number of abnormalities/patient	2.9	3.9	1.4 ^f^	

pPCL, primary plasma cell leukemia; sPCL, secondary plasma cell leukemia; NDMM, newly diagnosed Multiple Myeloma. ^a^ refers to differences between sPCL vs. NDMM; ^b^ refers to differences between pPCL vs. NDMM and between pPCL vs. sPCL; ^c^ refers to differences between sPCL vs. NDMM and between pPCL vs. sPCL; ^d^ refers to differences between pPCL vs. NDMM; ^e^ number of NDMM patients having tested for the whole panel of aberrations presented here; ^f^ based on 252 NDMM patients, tested for all aberrations presented here.

## Data Availability

The results of the study are available upon request from the corresponding author.

## Supplementary Materials

The following supporting information can be downloaded at: https://www.mdpi.com/article/10.3390/biomedicines10020209/s1, Figure S1: Schematic representation of 13q deletions in the three plasma cell dyscrasias; Table S1: List of probes used for the evaluation of cytogenetic abnormalities described in the study; Table S2: The cytogenetic pattern of t(11;14) in positive NDMM and pPCL patients examined with the two t(11;14) dual-fusion probes; Table S3: Genes hosted within the 11q13.3 locus found rearranged with the application of the t(11;14) dual fusion XT probe in three pPCL patients.

## References

[1] García-Sanz R., Orfão A., González M., Tabernero M.D., Bladé J., Moro M.J., Fernández-Calvo J., Sanz M.A., Pérez-Simón J.A., Rasillo A. (1999). Primary plasma cell leukemia: Clinical, immunophenotypic, DNA ploidy, and cytogenetic characteristics. Blood.

[2] Ramsingh G., Mehan P., Luo J., Vij R., Morgensztern D. (2009). Primary plasma cell leukemia: A surveillance, epidemiology, and end results database analysis between 1973 and 2004. Cancer.

[3] Gundesen M.T., Lund T., Moeller H.E.H., Abildgaard N. (2019). Plasma Cell Leukemia: Definition, Presentation, and Treatment. Curr. Oncol. Rep..

[4] Kyle R.A., Maldonado J.E., Bayrd E.D. (1974). Plasma cell leukemia. Report on 17 cases. Arch. Intern. Med..

[5] Gonsalves W.I., Rajkumar S.V., Gupta V., Morice W.G., Timm M.M., Singh P.P., Dispenzieri A., Buadi F.K., Lacy M.Q., Kapoor P. (2014). Quantification of clonal circulating plasma cells in newly diagnosed multiple myeloma: Implications for redefining high-risk myeloma. Leukemia.

[6] Granell M., Calvo X., Garcia-Guiñón A., Escoda L., Abella E., Martínez C.M., Teixidó M., Gimenez M.T., Senín A., Sanz P. (2017). Prognostic impact of circulating plasma cells in patients with multiple myeloma: Implications for plasma cell leukemia definition. Haematologica.

[7] Ravi P., Kumar S.K., Roeker L., Gonsalves W., Buadi F., Lacy M.Q., Go R.S., Dispenzieri A., Kapoor P., Lust J.A. (2018). Revised diagnostic criteria for plasma cell leukemia: Results of a Mayo Clinic study with comparison of outcomes to multiple myeloma. Blood Cancer J..

[8] Musto P., Simeon V., Martorelli M.C., Petrucci M.T., Cascavilla N., Di Raimondo F., Caravita T., Morabito F., Offidani M., Olivieri A. (2014). Lenalidomide and low-dose dexamethasone for newly diagnosed primary plasma cell leukemia. Leukemia.

[9] de Larrea C.F., Kyle R., Rosiñol L., Paiva B., Engelhardt M., Usmani S., Caers J., Gonsalves W., Schjesvold F., Merlini G. (2021). Primary plasma cell leukemia: Consensus definition by the International Myeloma Working Group according to peripheral blood plasma cell percentage. Blood Cancer J..

[10] Jurczyszyn A., Radocha J., Davila J., Fiala M.A., Gozzetti A., Grząśko N., Robak P., Hus I., Waszczuk-Gajda A., Guzicka-Kazimierczak R. (2018). Prognostic indicators in primary plasma cell leukaemia: A multicentre retrospective study of 117 patients. Br. J. Haematol..

[11] Suska A., Vesole D.H., Castillo J.J., Kumar S.K., Parameswaran H., Mateos M.V., Facon T., Gozzetti A., Mikala G., Szostek M. (2020). Plasma Cell Leukemia—Facts and Controversies: More Questions than Answers?. Clin. Hematol. Int..

[12] Papadimitriou K., Tsakirakis N., Malandrakis P., Vitsos P., Metousis A., Orologas-Stavrou N., Ntanasis-Stathopoulos I., Kanellias N., Eleutherakis-Papaiakovou E., Pothos P. (2020). Deep Phenotyping Reveals Distinct Immune Signatures Correlating with Prognostication, Treatment Responses, and MRD Status in Multiple Myeloma. Cancers.

[13] Tiedemann R.E., Gonzalez-Paz N., Kyle R.A., Santana-Davila R., Price-Troska T., Van Wier S.A., Chng W.J., Ketterling R.P., Gertz M.A., Henderson K. (2008). Genetic aberrations and survival in plasma cell leukemia. Leukemia.

[14] Katodritou E., Terpos E., Kelaidi C., Kotsopoulou M., Delimpasi S., Kyrtsonis M.-C., Symeonidis A., Giannakoulas N., Stefanoudaki A., Christoulas D. (2014). Treatment with bortezomib-based regimens improves overall response and predicts for survival in patients with primary or secondary plasma cell leukemia: Analysis of the Greek myeloma study group. Am. J. Hematol..

[15] Jurczyszyn A., Castillo J.J., Avivi I., Czepiel J., Davila J., Vij R., Fiala M.A., Gozzetti A., Grząśko N., Milunovic V. (2019). Secondary plasma cell leukemia: A multicenter retrospective study of 101 patients. Leuk. Lymphoma.

[16] Kostopoulos I.V., Eleutherakis-Papaiakovou E., Rousakis P., Ntanasis-Stathopoulos I., Panteli C., Orologas-Stavrou N., Kanellias N., Malandrakis P., Liacos C.-I., Papaioannou N.E. (2021). Aberrant Plasma Cell Contamination of Peripheral Blood Stem Cell Autografts, Assessed by Next-Generation Flow Cytometry, Is a Negative Predictor for Deep Response Post Autologous Transplantation in Multiple Myeloma; A Prospective Study in 199 Patients. Cancers.

[17] Kostopoulos I.V., Paterakis G., Papadimitriou K., Pavlidis D., Tsitsilonis O.E., Papadhimitriou S.I. (2015). Immunophenotypic analysis reveals heterogeneity and common biologic aspects in monoclonal B-cell lymphocytosis. Genes Chromosomes Cancer.

[18] Chretien M.-L., Corre J., Lauwers-Cances V., Magrangeas F., Cleynen A., Yon E., Hulin C., Leleu X., Orsini-Piocelle F., Blade J.-S. (2015). Understanding the role of hyperdiploidy in myeloma prognosis: Which trisomies really matter?. Blood.

[19] Samur A.A., Minvielle S., Shammas M., Fulciniti M., Magrangeas F., Richardson P.G., Moreau P., Attal M., Anderson K.C., Parmigiani G. (2019). Deciphering the chronology of copy number alterations in Multiple Myeloma. Blood Cancer J..

[20] Rajkumar S.V., Harousseau J.-L., Durie B., Anderson K.C., Dimopoulos M., Kyle R., Blade J., Richardson P., Orlowski R., Siegel D. (2011). International Myeloma Workshop Consensus Panel 1. Consensus recommendations for the uniform reporting of clinical trials: Report of the International Myeloma Workshop Consensus Panel 1. Blood.

[21] Flores-Montero J., Sanoja-Flores L., Paiva B., Puig N., García-Sánchez O., Böttcher S., Van Der Velden V.H.J., Pérez-Morán J.-J., Vidriales M.-B., García-Sanz R. (2017). Next Generation Flow for highly sensitive and standardized detection of minimal residual disease in multiple myeloma. Leukemia.

[22] Terpos E., Kostopoulos I.V., Kastritis E., Ntanasis-Stathopoulos I., Migkou M., Rousakis P., Argyriou A.T., Kanellias N., Fotiou D., Eleutherakis-Papaiakovou E. (2019). Impact of Minimal Residual Disease Detection by Next-Generation Flow Cytometry in Multiple Myeloma Patients with Sustained Complete Remission after Frontline Therapy. HemaSphere.

[23] Sanoja-Flores L., Flores-Montero J., Garcés J.J., Paiva B., Puig N., García-Mateo A., García-Sánchez O., Corral-Mateos A., Burgos L., Blanco E. (2018). (EuroFlow consortium) Next generation flow for minimally-invasive blood characterization of MGUS and multiple myeloma at diagnosis based on circulating tumor plasma cells (CTPC). Blood Cancer J..

[24] Tuazon S.A., Holmberg L.A., Nadeem O., Richardson P.G. (2021). A clinical perspective on plasma cell leukemia; current status and future directions. Blood Cancer J..

[25] Chang H., Sloan S., Li D., Patterson B. (2005). Genomic aberrations in plasma cell leukemia shown by interphase fluorescence in situ hybridization. Cancer Genet. Cytogenet..

[26] Chang H., Qi X., Yeung J., Reece D., Xu W., Patterson B. (2009). Genetic aberrations including chromosome 1 abnormalities and clinical features of plasma cell leukemia. Leuk. Res..

[27] Rotaru I., Găman G., Dumitrescu D., Foarfă C. (2012). Secondary plasma cell leukemia. Rom. J. Morphol. Embryol..

[28] Glavey S.V., Flanagan L., Bleach R., Kelly C., Quinn J., Ni Chonghaile T., Murphy P. (2020). Secondary plasma cell leukaemia treated with single agent venetoclax. Br. J. Haematol..

[29] Kupsh A., Arnall J., Voorhees P. (2019). A successful case of venetoclax-based therapy in relapsed/refractory secondary plasma cell leukemia. J. Oncol. Pharm. Pract..

[30] Mina R., D’Agostino M., Cerrato C., Gay F., Palumbo A. (2017). Plasma cell leukemia: Update on biology and therapy. Leuk. Lymphoma.

[31] Avet-Loiseau H., Daviet A., Brigaudeau C., Callet-Bauchu E., Terreé C., Lafage-Pochitaloff M., Deésangles F., Ramond S., Talmant P., Bataille R. (2001). Cytogenetic, interphase, and multicolor fluorescence in situ hybridization analyses in primary plasma cell leukemia: A study of 40 patients at diagnosis, on behalf of the Intergroupe Francophone du Myeélome and the Groupe Francçais de Cytogeéneétique Heématologique. Blood.

[32] Lionetti M., Musto P., Di Martino M.T., Fabris S., Agnelli L., Todoerti K., Tuana G., Mosca L., Cantafio M.E.G., Grieco V. (2013). Biological and Clinical Relevance of miRNA Expression Signatures in Primary Plasma Cell Leukemia. Clin. Cancer Res..

[33] Todoerti K., Agnelli L., Fabris S., Lionetti M., Tuana G., Mosca L., Lombardi L., Grieco V., Bianchino G., D’Auria F. (2013). Transcriptional Characterization of a Prospective Series of Primary Plasma Cell Leukemia Revealed Signatures Associated with Tumor Progression and Poorer Outcome. Clin. Cancer Res..

[34] Royer B., Minvielle S., Diouf M., Roussel M., Karlin L., Hulin C., Arnulf B., Macro M., Cailleres S., Brion A. (2016). Bortezomib, Doxorubicin, Cyclophosphamide, Dexamethasone Induction Followed by Stem Cell Transplantation for Primary Plasma Cell Leukemia: A Prospective Phase II Study of the Intergroupe Francophone du Myélome. J. Clin. Oncol..

[35] Lakshman A., Alhaj Moustafa M., Rajkumar S.V., Dispenzieri A., Gertz M.A., Buadi F.K., Lacy M.Q., Dingli D., Fonder A.L., Hayman S.R. (2018). Natural history of t(11;14) multiple myeloma. Leukemia.

[36] An G., Xu Y., Shi L., Zou D., Deng S., Sui W., Xie Z., Hao M., Chang H., Qiu L. (2013). t(11;14) multiple myeloma: A subtype associated with distinct immunological features, immunophenotypic characteristics but divergent outcome. Leuk. Res..

[37] Todoerti K., Taiana E., Puccio N., Favasuli V., Lionetti M., Silvestris I., Gentile M., Musto P., Morabito F., Gianelli U. (2021). Transcriptomic Analysis in Multiple Myeloma and Primary Plasma Cell Leukemia with t(11;14) Reveals Different Expression Patterns with Biological Implications in Venetoclax Sensitivity. Cancers.

[38] Janssen J.W., Vaandrager J.W., Heuser T., Jauch A., Kluin P.M., Geelen E., Bergsagel P.L., Kuehl W.M., Drexler H.G., Otsuki T. (2000). Concurrent activation of a novel putative transforming gene, myeov, and cyclin D1 in a subset of multiple myeloma cell lines with t(11;14)(q13;q32). Blood.

[39] Chiecchio L., Dagrada G.P., White H.E., Towsend M.R., Protheroe R.K., Cheung K.L., Stockley D.M., Orchard K.H., Cross N.C., Harrison C.J. (2009). Frequent upregulation of MYC in plasma cell leukemia. Genes Chromosomes Cancer.

[40] Fonseca R., Oken M.M., Harrington D., Bailey R.J., Van Wier S.A., Henderson K.J., Kay N.E., Van Ness B., Greipp P.R., Dewald G.W. (2001). Deletions of chromosome 13 in multiple myeloma identified by interphase FISH usually denote large deletions of the q arm or monosomy. Leukemia.

[41] Saxe D., Seo E.J., Bergeron M.B., Han J.Y. (2019). Recent advances in cytogenetic characterization of multiple myeloma. Int. J. Lab. Hematol..

[42] Pagano L., Valentini C.G., De Stefano V., Venditti A., Visani G., Petrucci M.T., Candoni A., Specchia G., Visco C., Pogliani E.M. (2011). Primary plasma cell leukemia: A retrospective multicenter study of 73 patients. Ann. Oncol..

[43] Schinke C., Boyle E.M., Ashby C., Wang Y., Lyzogubov V., Wardell C., Qu P., Hoering A., Deshpande S., Ryan K. (2020). Genomic analysis of primary plasma cell leukemia reveals complex structural alterations and high-risk mutational patterns. Blood Cancer J..

[44] Keats J.J., Chesi M., Egan J.B., Garbitt V.M., Palmer S.E., Braggio E., Van Wier S., Blackburn P.R., Baker A.S., Dispenzieri A. (2012). Clonal competition with alternating dominance in multiple myeloma. Blood.

[45] Egan J.B., Shi C.-X., Tembe W., Christoforides A., Kurdoglu A., Sinari S., Middha S., Asmann Y., Schmidt J., Braggio E. (2012). Whole-genome sequencing of multiple myeloma from diagnosis to plasma cell leukemia reveals genomic initiating events, evolution, and clonal tides. Blood.

[46] Jones J.R., Weinhold N., Ashby C., Walker B.A., Wardell C., Pawlyn C., Rasche L., Melchor L., Cairns D.A., Gregory W.M. (2019). Clonal evolution in myeloma: The impact of maintenance lenalidomide and depth of response on the genetics and subclonal structure of relapsed disease in uniformly treated newly diagnosed patients. Haematologica.

[47] Locher M., Steurer M., Jukic E., Keller M.A., Fresser F., Ruepp C., Wöll E., Verdorfer I., Gastl G., Willenbacher W. (2020). The prognostic value of additional copies of 1q21 in multiple myeloma depends on the primary genetic event. Am. J. Hematol..

[48] Yan Y., Qin X., Liu J., Fan H., Yan W., Liu L., Du C., Yu Z., Xu Y., Hao M. (2021). Clonal phylogeny and evolution of critical cytogenetic aberrations in multiple myeloma at single cell level by QM-FISH. Blood Adv..

[49] Mangiacavalli S., Pochintesta L., Cocito F., Pompa A., Bernasconi P., Cazzola M., Corso A. (2013). Correlation between burden of 17P13.1 alteration and rapid escape to plasma cell leukaemia in multiple myeloma. Br. J. Haematol..

[50] Jovanović K.K., Escure G., Demonchy J., Willaume A., Van De Wyngaert Z., Farhat M., Chauvet P., Facon T., Quesnel B., Manier S. (2019). Deregulation and Targeting of TP53 Pathway in Multiple Myeloma. Front. Oncol..

[51] Kostopoulos I.V., Paterakis G., Pavlidis D., Kastritis E., Terpos E., Tsitsilonis O.E., Papadhimitriou S.I. (2017). Clonal evolution is a prognostic factor for the clinical progression of monoclonal B-cell lymphocytosis. Blood Cancer J..

[52] Binder M., Rajkumar S.V., Ketterling R.P., Dispenzieri A., Lacy M.Q., Gertz M.A., Buadi F.K., Hayman S.R., Hwa Y.L., Zeldenrust S.R. (2016). Occurrence and prognostic significance of cytogenetic evolution in patients with multiple myeloma. Blood Cancer J..

[53] Colović M., Janković G., Suvajdzić N., Milić N., Dordević V., Janković S. (2008). Thirty patients with primary plasma cell leukemia: A single center experience. Med. Oncol..

[54] Mosca L., Musto P., Todoerti K., Barbieri M., Agnelli L., Fabris S., Tuana G., Lionetti M., Bonaparte E., Sirchia S.M. (2013). Genome-wide analysis of primary plasma cell leukemia identifies recurrent imbalances associated with changes in transcriptional profiles. Am. J. Hematol..

[55] Jung S.H., Lee J.J., Kim K., Suh C., Yoon D.H., Min C.K., Sohn S.K., Choi C.W., Lee H.S., Kim H.J. (2017). Korean Multiple Myeloma Working Party. The role of frontline autologous stem cell transplantation for primary plasma cell leukemia: A retrospective multicenter study (KMM160). Oncotarget.

