# Supplementary material for: The Cytogenetic Profile of Primary and Secondary Plasma Cell Leukemia: Etiopathogenetic Perspectives, Prognostic Impact and Clinical Relevance to Newly Diagnosed Multiple Myeloma with Differential Circulating Clonal Plasma Cells
Source: Biomedicines. 2022 Jan 19;10(2):209. doi: 10.3390/biomedicines10020209 (PMC8869452; doi:10.3390/biomedicines10020209)
Supplement: Supplementary file 1 [file biomedicines-10-00209-s001.zip › biomedicines-1501487-supplementary.pdf]

Supplementary Materials

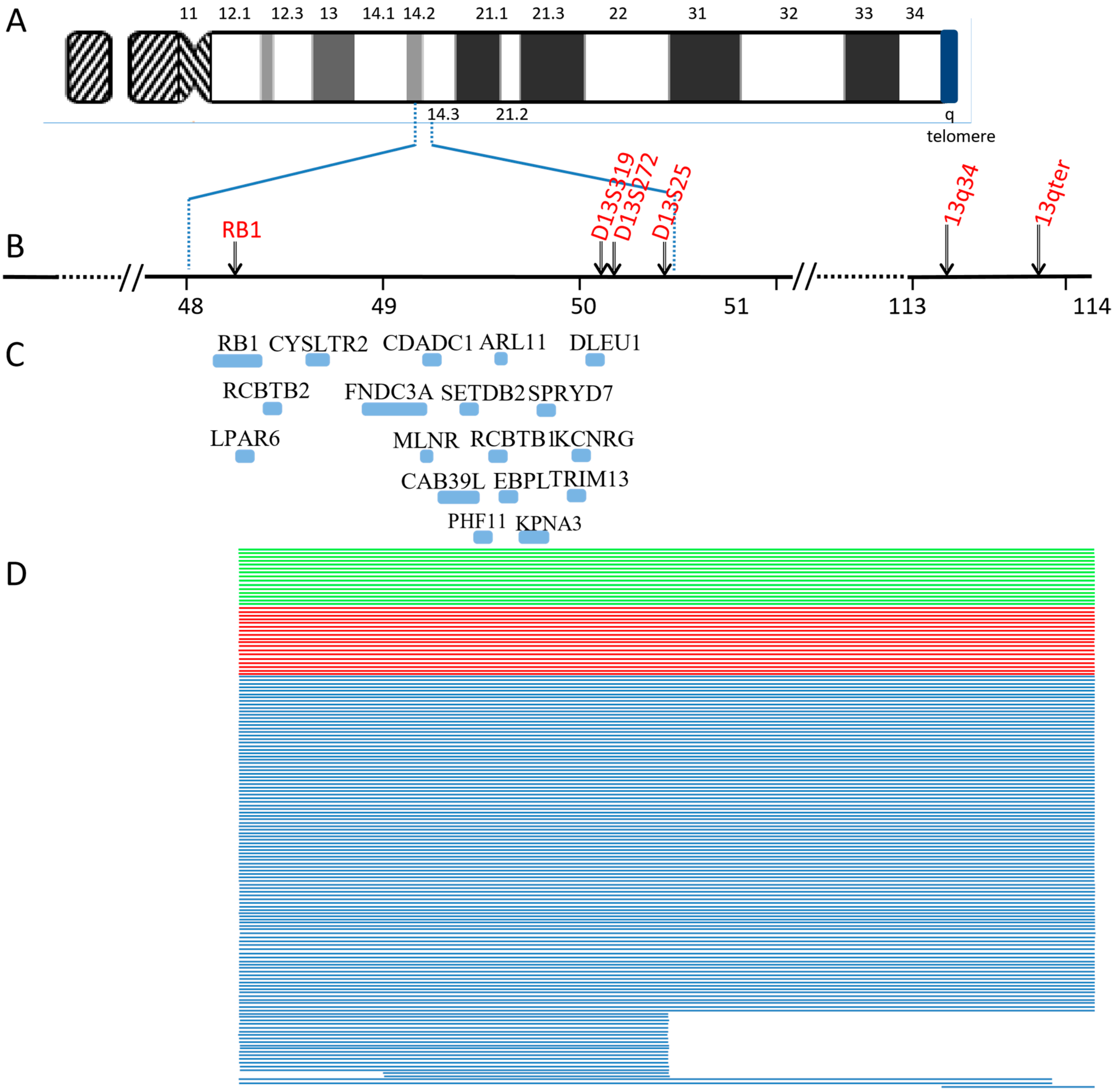

Figure S1. Schematic representation of 13q deletions in the three plasma cell dyscrasias. **A**: Ideogram of chromosome 13. **B**: Physical mapping of the six utilized 13q FISH probes. **C**: Coding genes in the 13q14 locus which consisted the minimal deleted region in all but one cases described here; **D**: The extend of 13q deletion for all NDMM and PCL patients with evidence of 13q deletion. Each line represents a single patient with green color corresponding to pPCL, red to sPCL and blue to NDMM patients respectively.

Table S1: List of probes used for the evaluation of cytogenetic abnormalities described in the study.

Abnormality	Probe Name	Cytogenic Location	Cut-Off
13q-	LSI 13 RB1 (13q14)	13q14.2	8%
13q-	LSI D13S25 (13q14.3)	13q14.3	8%
13q-	LSI D13S272 (13q14.3)	13q14.3	8%
13q-	LSI D13S319 (13q14.3)	13q14.3	8%
13q-	LSI 13q34	13q34	8%
13q-	TelVysion 13q	VIJyRM2002 (D13S327)	8%
t(11;14)(q13;q32)	LSI IGH/CCND1 DF	11q13.3/14q32.3	2F1G1R (3%)<br>1F2G1R (5%)
t(11;14)(q13;q32)	LSI IGH/CCND1 XT DF	11q13.3/14q32.3	2F1G1R (3%)
t(4;14)(p16;q32)	LSI IGH/FGFR3 Dual-Color DF	4p16.3/14q32.3	2F1G1R (3%)
t(14;16)(q32;q23)	LSI IGH/MAF DF	14q32.3/16q23	2F1G1R (3%)
t(8;14)(q24;q32)	LSI IGH/MYC/CEP 8 Tri-color DF	14q32.3/8q24.2/8p11.1-q11.1	2F1G1R (3%)
t(8q24)	LSI MYC Break apart	8q24.21	4%
+1q21/ del(1p32)	LSI CKS1B/LSI CDKN2C	1q21.2/1p32.3	+1q21 (4%)<br>del(1p32) (8%)
del(17p13)/-17	LSI TP53/ CEP17	17p13.1/17p11.1-q11.1	-17 (4%)<br>del(17p13) (4%)
Hyperdiploidy	LSI D5S23,D5S721/CEP9/CEP15	5p15.2/9p11-q11/15p11.1-q11.1	4% *

Abbreviations: LSI: Locus Specific Identifier; CEP: Chromosome Enumeration Probe; DF: Dual Fusion. * For more than 3 signals from any probe.

Table S2. The cytogenetic pattern of t(11;14) in positive NDMM and pPCL patients examined with the two t(11;14) dual-fusion probes.

Patient	Signal Formation	Possible interpretation
MM 1	2F 1G 1R	Typical
MM 2	1F 1G 1R	Insertion of IGH in 11q13 or deletion of der(14) or der(11)
MM 3	2F 1G 1R	Typical
MM 4	2F 1G 1R	Typical
MM 5	2F 1G 1R	Typical
MM 6	3F 1G 1R	Duplication of der(14) due to HD
MM 7	2F 1G 1R	Typical
MM 8	2F 1G 1R	Typical
MM 9	4F 2G 2R	Duplication of der(11), duplication of der(14) and duplication of non-rearranged chromosomes 11 and 14 due to HD
MM 10	3F 2G 1R	Duplication of der(14) and t(14q32) of the other allele, other than t(4;14) and t(14;16)
MM 11	1F 2G 1R /2F 1G 1R	Non typical 11q13 breakpoint. The second fusion signal was detectable only with the larger CCND1 XT probe
MM 12	2F 1G 1R	Typical
MM 13	2F 1G 1R	Typical
MM 14	4F 1G 1R	Duplication of der (11) and der(14) due to HD
MM 15	2F 1G 1R	Typical
MM 16	1F 2G 1R /2F 1G 1R	Non typical 11q13 breakpoint. The second fusion signal was detectable only with the larger CCND1 XT probe
MM 17	2F 1G 1R	Typical
MM 18	2F 1R	Monosomy of Chromosome 14
MM 19	2F 1G 1R	Typical
MM 20	2F 1G 1R	Typical
MM 21	2F 1G 1R	Typical
MM 22	3F 2G 2R	Duplication of der(14) and duplication of non-rearranged chromosomes 11 and 14 due to HD
MM 23	2F 1G 1R	Typical
MM 24	2F 1G 2R	Duplication of non-rearranged chromosome 11
MM 25	1F 1G 2R	Loss of VH segments due to VDJ recombination
MM 26	2F 1G 1R	Typical
MM 27	1F 2G 1R /2F 1G 1R	Non typical 11q13 breakpoint. The second fusion signal was detectable only with the larger CCND1 XT probe
MM 28	2F 1G 1R	Typical
MM 29	2F 1G 1R	Typical
MM 30	1F 2G 1R /2F 1G 1R	Non typical 11q13 breakpoint. The second fusion signal was detectable only with the larger CCND1 XT probe
MM 31	2F 1G 1R	Typical
MM 32	3F 1G 1R	Duplication of der(14) due to HD
MM 33	2F 1G 1R	Typical
MM 34	3F 1G 1R	Duplication of der(14) due to HD
MM 35	2F 1G 1R	Typical
MM 36	1F 1G 2R	Loss of VH segments due to VDJ recombination
MM 38	1F 2G 1R /2F 1G 1R	Non typical 11q13 breakpoint. The second fusion signal was detectable only with the larger CCND1 XT probe
MM 39	2F 1G 1R	Typical
MM 40	2F 1G 1R	Typical
MM 40	2F 1G 1R	Typical

pPCL 1	2F 1G 1R	Typical
pPCL 2	1F 2G 1R /2F 1G 1R	Non typical 11q13 breakpoint. The second fusion signal was detectable only with the larger CCND1 XT probe
pPCL 3	2F 1G 1R	Typical
pPCL 4	2F 1G 1R	Typical
pPCL 5	1F 2G 1R /2F 1G 1R	Non typical 11q13 breakpoint. The second fusion signal was detectable only with the larger CCND1 XT probe
pPCL 6	2F 2G 1R /3F 1G 1R	Non typical 11q13 breakpoint and duplication of der(11). The additional fusion signal (on der(14)] was detectable only with the larger CCND1 XT probe
pPCL 7	2F 1G 1R	Typical
pPCL 8	1F 2G 1R /2F 1G 1R	Non typical 11q13 breakpoint. The second fusion signal was detectable only with the larger CCND1 XT probe
pPCL 9	3F 1G 1R	Dupl of der(14) due to HD
pPCL 10	2F 1G 1R	Typical
pPCL 11	1F 2G 1R /2F 1G 1R	Non typical 11q13 breakpoint. The second fusion signal was detectable only with the larger CCND1 XT probe
pPCL12	2F 1G 1R	Typical
pPCL13	1F 2G 1R /2F 1G 1R	Non typical 11q13 breakpoint. The second fusion signal was detectable only with the larger CCND1 XT probe

Table S3. Genes hosted within the 11q13.3 locus found rearranged with the application of the t(11;14) dual fusion XT probe in three pPCL patients.

Gene	Name/Biotype	Entrez Gene ID/ Ensembl ID	Genomic Location *	Related Pathways	Gene Ontology
RP11-757G1.6	lincRNA	ENSG00000250508	68,870,664-68,874,542	No data available	No data available
MRPL21	Mitochondrial Ribosomal protein L21	ENSG00000197345	68,891,276-68,903,835	i) Organelle biogenesis and maintenance ii) Mitochondrial translation iii) Viral mRNA translation	Structural constituent of ribosome
IGHMBP2	Immunoglobulin Mu Binding Protein 2	ENSG00000132740	68,903,842-68,940,602	No data available	Nucleid acid binding, DNA helicase activity
RP11-757G1.5	antisence lncRNA	ENSG00000255741	68,941,503-68,942,852	No data available	No data available
MRGPRD	Mas Related GPR Family Member D	ENSG00000172938	68,980,021-68,980,986	Angiotensin-converting-enzyme inhibitor pathway, Pharmacodynamics	G-protein coupled receptor activity
RP11-554A11.4	Sense overlapping lncRNA	ENSG00000261625	69,000,765-69,002,048	No data available	No data available
RP11-554A11.5	Antisense lncRNA	ENSG00000261276	69,004,394-69,005,100	No data available	No data available
MRGPRF	Mas Related GPR Family Member F	ENSG00000172935	69,004,394-69,013,409	No data available	G-protein coupled receptor activity
MRGPRF-AS1	Mas Related GPR Family Member F antisense RNA 1	ENSG00000256508	69,012,283-69,018,447	No data available	No data available
TPCN2	Two Pore Segment Channel 2	ENSG00000162341	69,048,882-69,162,440	i) Ion channel transport ii) Pancreatic secretion iii) Transmembrane transport of small molecules (glucose, bile salts and organic acids, metal ions and amine compounds)	Identical protein binding, ion channel activity, calcium ion homeostasis, smooth muscle contraction, lysosome organization
MIR3164	microRNA 3164	ENSG00000265539	69,083,176-69,083,258	No data available	No data available
RP11-554A11.7	lincRNA	ENSG00000260895	69,103,493-69,109,094	No data available	No data available
RP11-554A11.8	lincRNA	ENSG00000261070	69,147,228-69,171,564	No data available	No data available
RP11-554A11.9	antisense lncRNA	ENSG00000259799.1	69,155,910-69,159,752	No data available	No data available

* According to GRCh38/hg38 coordinates.
